# Biases in object location estimation: The role of rotations and translation

**DOI:** 10.3758/s13414-023-02716-2

**Published:** 2023-05-31

**Authors:** Vladislava Segen, Marios N. Avraamides, Timothy Slattery, Jan M. Wiener

**Affiliations:** 1 Aging and Dementia Research Centre, Bournemouth University, Poole, UK; 2 Department of Psychology, Bournemouth University, Poole, UK; 3 German Centre for Neurodegenerative Disease, Magdeburg, Germany; 4 Department of Psychology, University of Cyprus, Nicosia, Cyprus; 5 CYENS Centre of Excellence, Nicosia, Cyprus

**Keywords:** Spatial perspective-taking, Spatial memory, Object-location memory, Spatial cognition

## Abstract

Spatial memory studies often employ static images depicting a scene, an array of objects, or environmental features from one perspective and then following a perspective-shift-prompt memory either of the scene or objects within the scene. The current study investigated a previously reported systematic bias in spatial memory where, following a perspective shift from encoding to recall, participants indicated the location of an object farther to the direction of the shift. In Experiment 1, we aimed to replicate this bias by asking participants to encode the location of an object in a virtual room and then indicate it from memory following a perspective shift induced by camera translation and rotation. In Experiment 2, we decoupled the influence of camera translations and rotations and examined whether adding additional objects to the virtual room would reduce the bias. Overall, our results indicate that camera translations result in greater systematic bias than camera rotations. We propose that the accurate representation of camera translations requires more demanding mental computations than camera rotations, leading to greater uncertainty regarding the location of an object in memory. This uncertainty causes people to rely on an egocentric anchor, thereby giving rise to the systematic bias in the direction of camera translation.

**Supplementary Information:**

The online version contains supplementary material available at 10.3758/s13414-023-02716-2.

Our ability to navigate the environment effectively is closely tied to our ability to recognize our surroundings and the places we find ourselves in. This ability, known as place recognition, is dependent on our capacity to remember the locations of objects in relation to one another, as well as our ability to retrieve this information from different perspectives (Epstein et al., 1999; Postma et al., 2004; Waller, 2006). Self-motion information during travel allows updating the representation of a place to match the new point of view (Waller et al., 2002). However, in the absence of self-motion information, recognition across different perspectives is achieved by mentally manipulating a representation, a process known as spatial perspective-taking (Holmes et al., 2018; King et al., 2002; Klencklen et al., 2012). Such reasoning is frequent in everyday life (e.g., finding our way by inspecting a misaligned You Are Here map, providing route directions to a tourist in our city, recognizing places that we approach from novel directions).

Place recognition is typically studied with spatial perspective-taking tasks in which participants are presented with static images depicting a scene, an array of objects, or environmental features from one perspective and are then asked to indicate whether the array has changed when presented from a different perspective (Diwadkar & McNamara, 1997; Hartley et al., 2007; Hilton et al., 2020; Montefinese et al., 2015; Muffato et al., 2019; Schmidt et al., 2007; Segen, Avraamides, Slattery, & Wiener, 2021c, 2021d; Sulpizio et al., 2013).

Our recent research suggests that such paradigms may yield a systematic bias in reporting memorized object locations (Segen et al., 2022; Segen, Avraamides, Slattery, Colombo, & Wiener, 2021a; Segen, Colombo, Avraamides, Slattery, & Wiener, 2021b). Specifically, we found that when participants were asked to indicate the location of an object following a perspective shift (Segen et al., 2022; Segen et al., 2021a) or when asked to judge the direction in which an object moved after a perspective shift (Segen et al., 2021b), they systematically localized the object further in the direction of the perspective shift. Interestingly, this systematic bias in the direction of the perspective shift is not driven by distortions introduced in memory as participants also exhibited this bias in a perceptual version of the task (Segen et al., 2022).

Our conjecture is that the perspective shift-related bias is caused by egocentric influences on target object estimates (Segen et al., 2022; Segen et al., 2021b). If participants relied solely on an allocentric representation in which the object position is encoded relative to other features in the environment, their own position and movement in the environment should not influence their responses. Consecutively, presence of a perspective shift should therefore not result in systematic biases (Ekstrom, Arnold & Iaria, 2014). Thus, in line with recent research showing that participants are more likely to rely on egocentric rather than allocentric representations of object locations for small perspective shifts (Heywood-Everett et al., 2022), we propose that participants in our task rely on an egocentric representation that biases their memory for the location of the object towards the egocentric vector to the object experienced during encoding (i.e., before the perspective shift). Specifically, we propose that the uncertainty about the exact nature of the perspective shift increases the uncertainty about the exact location of the object, resulting in a bias in object location estimates towards the egocentric self-to-object vector derived during encoding. This idea aligns well with the anchoring and adjustment heuristic proposed by Tversky and Kahneman (1974), according to which people base their responses on initial estimates (the anchor) that they adjust to correct for errors when they are uncertain. Often, these anchors are based on egocentric representations (Epley et al., 2004; Gilovich et al., 2000; Keysar et al., 2000). Epley et al. (2004), for example, found that participants’ prior exposure either to positive or negative events influenced their decision on whether others would judge ambiguous events as either sarcastic or genuine.

In our previous work (Segen et al., 2022; Segen et al., 2021a; Segen et al., 2021b), participants may have used the egocentric self-to-object vector as an anchor for their response, which would result in participants “dragging” the object with them following a perspective shift. In line with this explanation, previous research suggests that adjustments require time and cognitive effort (Epley et al., 2004) and as a result, individuals often stop adjusting their responses once a plausible estimate is reached thereby biasing their responses in the direction of the initial anchor (Quattrone, 1982; Tversky & Kahneman, 1974). If participants in our previous studies (Segen et al., 2022; Segen et al., 2021a) also showed insufficient adjustments, this would explain the systematic bias in the direction of the perspective shift.

Potential sources of uncertainty that could encourage the use of an egocentric anchor include (1) the uncertainty about the location of the object in the environment and (2) difficulties in understanding the exact nature of the perspective shift. The uncertainty about an object’s location could be reduced by enhancing the environment to include further spatial information (i.e., by adding stable environmental cues that help participants to encode the object’s location more accurately; Cánovas et al., 2011; Chamizo et al., 2011; Ekstrom & Yonelinas, 2020; Kamil & Cheng, 2001; Luo et al., 2007). Such cues may also improve the understanding of the perspective shifts. For example, participants can use the change in the egocentric relations to those cues as well as the changes in the visual projection of the cues to compute how their own position in space has changed following a perspective shift. Thus, we hypothesize that enriching the environment with further spatial information will reduce the uncertainty about the object’s location after the perspective shift and decrease the *perspective shift-related bias*.

It is also possible that the uncertainty about the perspective shift arises due to the way we introduced perspective shifts. For example, in our previous studies, the perspective shift consisted of a combination of camera translation and rotation movements (Segen et al., 2022; Segen et al, 2021a, 2021b). Specifically, the camera moved in a circle such that it translated in one direction and at the same time rotated in the opposite direction. This combination of camera translation and rotation is typical for spatial perspective-taking tasks (Hilton et al., 2020; Montefinese et al., 2015; Muffato et al., 2019; Schmidt et al., 2007; Segen et al., 2021c, 2021d; Sulpizio et al., 2013) as it allows for greater overlap between the parts of the environment that are visible before and after the perspective shift. Given the small perspective shifts introduced in our previous studies reporting the *perspective shift-related bias* (i.e., small translations requiring only 20° to 30° rotations), the resulting images looked quite similar. This may have produced difficulties in understanding the perspective shifts, increasing participants’ uncertainty regarding their simulated movement within the environment. For example, if participants thought that the camera movement between encoding and test was smaller than it actually was, this could have caused a bias in the direction of the perspective shift.

So far, the unique role of camera rotations and translations during perspective-taking has not been studied. Although our previous research suggests that the observed *perspective shift-related bias* is linked to the introduction of camera movements during perspective shifts, it is not clear whether it is driven by camera translations or rotations separately or by a specific combination of the two. Therefore, the main aim of this study is to investigate the contribution of camera rotations and translations to the p*erspective shift-related bias* that was observed in our previous work (Segen et al., 2022; Segen et al., 2021a, 2021b).

Although no earlier studies investigated the role of translations and rotations for perspective-taking separately, this has been done in tasks assessing spatial updating based on real or imagined body movements (Easton & Sholl, 1995; Presson & Montello, 1994; Rieser, 1989; Wraga, 2003). In such tasks, participants memorize an array of objects and are then asked to move or to imagine moving to a different location in the array and point to one of the objects from that new location.

With physical movement, there is no difference in performance when the new location is reached either by translation or rotation. In such cases, visual, vestibular and proprioceptive inputs during active movement are used to update the representation to allow place recognition from a different perspective (Waller et al., 2002). However, when participants are asked to imagine moving to a new location, rotations lead to greater errors and longer response times than translations (Avraamides et al., 2013; Easton & Sholl, 1995; Presson & Montello, 1994; Rieser, 1989; Sancaktar & Demirkan, 2008). Difficulties with imagined rotations are also highlighted by difficulties in using maps that are misaligned with participants’ orientation in space (Levine et al., 1982; Presson & Hazelrigg, 1984; Roskos-Ewoldsen et al., 1998). It is, however, not clear whether or how these results translate to spatial perspective-taking tasks in which participants do not need to “imagine” rotations or translations but must instead use the available information to determine how they have moved in space. The current study will address this issue.

## Aims and hypotheses

In the current study we conducted two experiments. The key aim of Experiment 1 was to provide a conceptual replication of Segen et al. (2022) in which we found a *perspective shift-related bias* during object location estimates. The key aim of Experiment 2 was to investigate the contribution of camera rotations and translations to the *perspective shift-related bias*. To do so, we manipulated camera rotation and the translations independently during the perspective shifts. We also investigated if enriching the environment with additional objects would improve participants’ ability to remember the precise locations of the target object across different camera rotations and translations. Specific predictions are presented in the introduction of the individual experiments.

## Experiment 1

### Introduction

In Experiment 1, we used a modified version of a task used in Segen et al. (2022) to investigate spatial memory across different perspectives. In the original task (Segen et al., 2022), participants memorized the locations of the target object that was always located on a plank in a virtual room. Then, following a short delay and a perspective shift, the target object disappeared, and participants were asked to indicate its location by selecting one of several predefined locations. In the current study, we introduced two key changes compared with the original task. First, we have removed the plank, which may have acted as an influential cue that restricted the possible locations where the target object could be placed. Second, participants’ responses were unconstrained. That is, instead of presenting participants with predefined object locations that were overlaid on the plank during the test phase, we asked them to indicate the location of the target object anywhere in the environment. Removing the plank and the location markers reduced the risk that participants relied on strategies we did not control for, and which could be responsible for the perspective shift-related bias.

The main aim of this experiment was to provide a conceptual replication of the results reported in Segen et al. (2022). Thus, we predicted that participants’ estimates of object locations would be biased in the direction of the perspective shift.

### Method

#### Participants

Twenty-eight participants between 18 and 35 years of age (mean age =24.04 years, *SD* = 4.69; age range: 18–33 years; 16 females and 12 males) took part in this study. Participants were recruited through the participant recruitment system of Bournemouth University and received course credit for their participation. All participants gave their informed consent in accordance with the Declaration of Helsinki (World Medical Association, 2004).

#### Materials

**Fig. 1:**
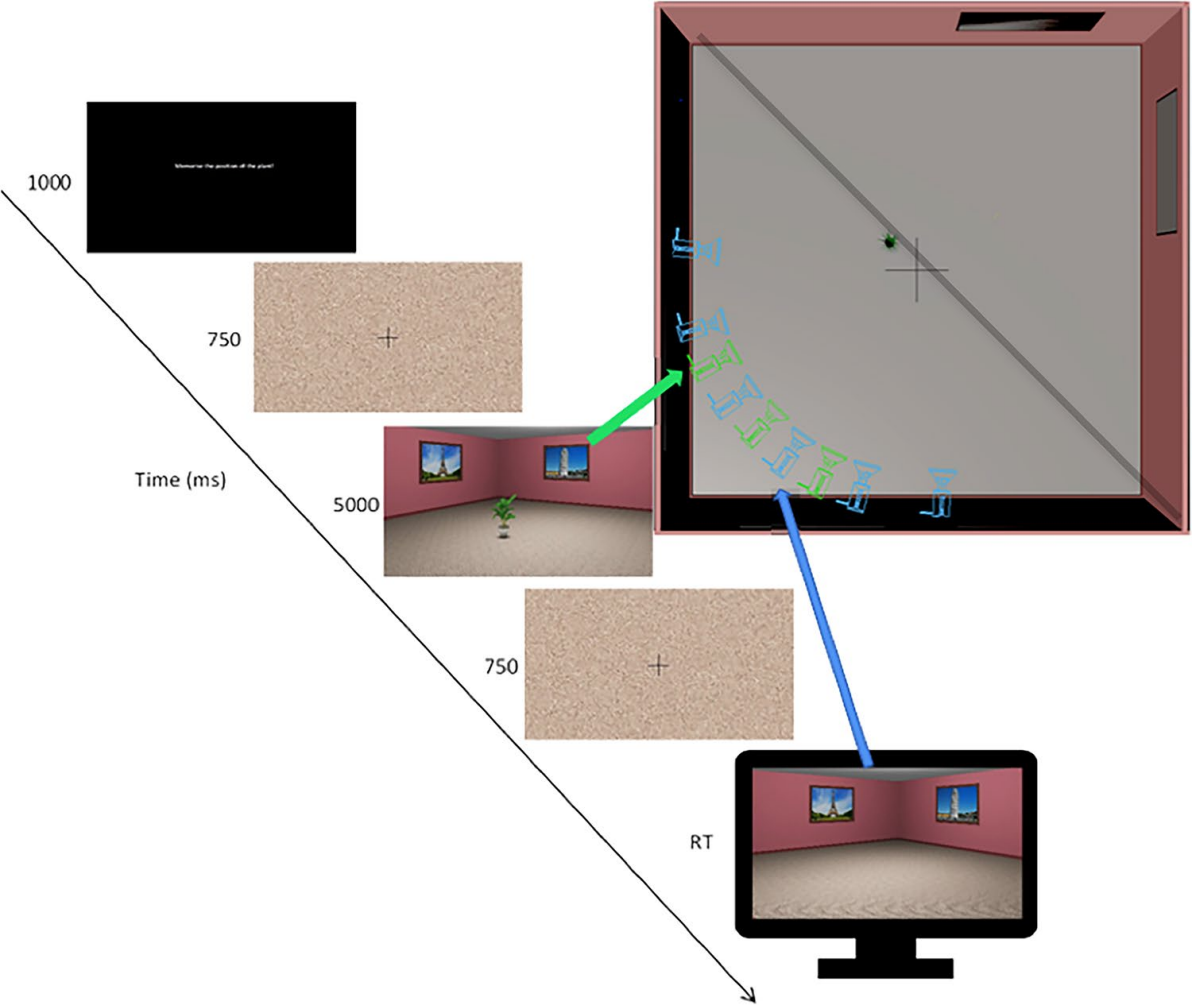
Top-down schematic of the virtual environment used in the experiment with camera locations (green and blue cameras represent camera locations at encoding and test, respectively). Black diagonal line represents the invisible diagonal line along which target objects were positioned. Trial structure with green and blue arrows indicating the cameras used to render the encoding and test scenes, respectively. (Colour figure online)

The virtual environment was designed with 3DS Max 2018 (Autodesk) and consisted of a square 9.8-m × 9.8-m room. Posters depicting famous landmarks were placed on the walls of the virtual room. The landmarks were chosen based on high familiarity ratings (Hamburger & Röser, 2014). The target object, a potted plant, was placed in one of 18 predefined locations and the scene for encoding was rendered from one of three camera locations (Fig. 1). The objects positions where the same as in Segen et al. (2022) and were arranged along a diagonal line in the middle of the room. The individual object positions were 14, 28, 42, 84, 98, 112, 168, 182, and 192 cm to the left or to the right of the centre of “invisible” diagonal line.

At test, the object was removed, and the scene was rendered from one of the six test camera locations (Fig. 1) such that there was a 30° perspective shift either to the left or to the right of the encoding location. The experimental stimuli were renderings of the environment with a 58° horizontal field of view. A custom asymmetric viewing frustum that resembles natural vision with a 15% shift in the vertical field of view was used. This asymmetric viewing frustum resembles natural vision and has been found to improve distance perception in virtual environments (Franz, 2005).

#### Procedure

The experiment was carried out online using Testable (testable.org). At the beginning of the experiment, participants were asked to adjust the screen zoom settings to ensure that the entire scene was visible during the experiment which was run in full-screen mode. Each experimental trial started with instructions to remember the location of the object (1,000 ms), followed by a fixation cross, and a scrambled stimuli mask presented for 750 ms (Fig. 1). In the encoding phase, participants were presented with a rendering of the room with one of the 18 possible target object locations from one of three encoding camera locations for 5 seconds. This was followed by the presentation of a fixation cross and a scrambled stimuli mask for 750 ms. Finally, in the test phase, participants were presented with a rendering of the room without the target object from one of the six possible camera locations (Fig. 1). Participants had to indicate the location of the object taking into account the camera movements between encoding and test. Participants moved the mouse cursor to the location where they thought the object was during encoding and clicked to register their response. They were instructed to use the base of the target object to remember the location it occupied on the floor.

Each of the 18 possible target object locations was presented twice for each of the three encoding camera locations which resulted in 108 experimental trials that took around 25 minutes to complete.

### Results

**Fig. 2:**
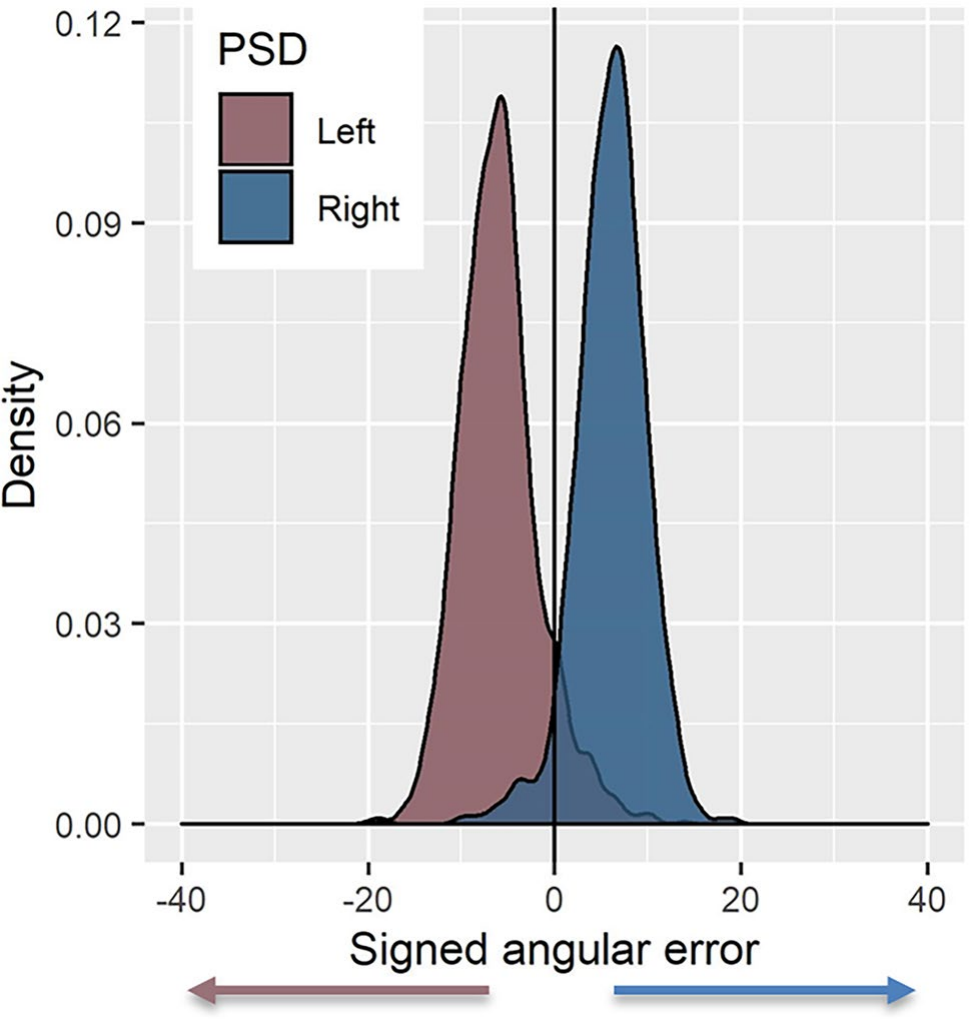
Distribution of signed angular error (°) as a function of PSD (left/right). Negative error indicates error to the left, and positive error indicates error to the right of the correct object location. (Colour figure online)

Since the main aim of this experiment was to investigate biases in the direction in which participants estimate object locations, for brevity, only signed angular error is reported. Distance errors are presented in the Supplementary Materials. Signed angular error is calculated by subtracting the angle between the participants response (estimated object location) and the camera position (i.e., participant position) from the angle between the camera position and the “correct” object position. Positive and negative angular error indicate that the object was estimated to be to the right or the left (respectively) of the correct object location. We ran linear mixed-effects models (LME) using LME4 (Bates et al., 2015) in R (R Core Team, 2013) to investigate the role perspective shift direction (PSD) had on signed angular error. PSD (left/right) was included as a fixed effect and was coded using sum contrasts such that left perspective shifts were compared with the average error for the left and right PSD. The LME also included a by-subject and by-item (unique stimuli [combination of object start locations and test camera]) random intercepts. We found that PSD (*left*) influenced participants’ error (β = −6.712, *SE* = 0.426, *t* = −15.743), with participants positioning the target object further to the left when the perspective shift was to the *left*. If we reverse the contrasts such that *right* PSD is compared with the grand average, a reverse pattern is found with participants’ error shifted to the right for *right* PSD. In other words, participants exhibited a bias in their estimates that were in the same direction as that of the perspective shift between encoding and test (Fig. 2).

### Discussion

Experiment 1 showed that when indicating target object locations, participants systematically made errors in the same direction as the perspective shift. The presence of a systematic shift in the estimates of the location of the target object in the same direction as the perspective shift, provides a conceptual replication of our previous findings (Segen et al., 2022; Segen, Avraamides, Slattery, Colombo, & Wiener, 2021a). Notably, in the original task, the objects were always placed on a plank and participants were provided with a set of predefined location markers on the plank to select from in order to indicate the location of the target object. In the current task, we removed both the plank and the location markers to rule out the possibility that these cues were related to the *perspective shift-related bias*. Thus, the presence of a systematic influence of the perspective shift on participants’ object location estimates in the current study suggests that the bias is driven by camera movements in the environment. In Experiment 2, we further explore the factors contributing to the *perspective shift-related bias*.

## Experiment 2

### Introduction

It is possible that the camera movements used in Experiment 1 and in other studies with spatial perspective tasks (Hilton et al., 2020; Montefinese et al., 2015; Muffato et al., 2019; Segen et al., 2021a, 2021b, 2021c, 2021d; Segen et al., 2022; Sulpizio et al., 2013) contributed to the *perspective shift-related bias* in target object location estimates. Specifically, we speculated that there might be something special about this combination of camera rotations and translations, where the camera translates in one direction and rotates in the opposite direction, which gives rise to the *perspective shift-related bias*. For example, participants may have difficulties in correctly perceiving the size of the perspective shift since the images rendered from both perspectives look strikingly similar. This is because the rotation in the opposite direction to the translation ensures that the same features of the scene remained visible. This could lead participants to systematically underestimate the extent of the camera movement and, thus lead to the systematic shift in the errors in direction of the camera shift.

The key aim of Experiment 2 was, therefore, to investigate the contribution of camera rotations and translations to the *perspective shift-related bias*. To do so, we varied camera rotations and translations independently by creating situations with rotations but without translations and vice versa. In addition, we introduced camera movements that we and others have used in previous work (Hilton et al., 2020; Montofinese et al., 2015; Muffato et al., 2019; Segen et al., 2021a, 2021b, 2021c, 2021d; Segen et al., 2022; Sulpizio et al., 2013), where the camera translates and rotates in opposite directions (*incongruent*), to investigate if only this specific combination of camera movements gives rise to the *perspective shift-related bias*. Lastly, we added a condition where the camera translates and rotates in the same direction.

To our knowledge, this was the first study using spatial perspective-taking in which camera rotations and translations are decoupled. We, therefore, had no specific prediction on how the camera movements would contribute to performance and the perspective shift-related bias. It is possible that participants would be more affected by camera rotations, as previous research on spatial updating shows that imagined rotations are harder than imagined translations (Easton & Sholl, 1995; Presson & Montello, 1994; Rieser, 1989; Sancaktar & Demirkan, 2008). Alternatively, if the *perspective shift-related bias* that we reported in earlier studies was driven by the specific camera movements that we have used where the rotation is always in a different direction to the translation, we would expect the bias to be present only in such situations.

In our previous work, we proposed that uncertainty about the location of the target object following perspective shift is likely to contribute to the *perspective shift-related bias* (Segen et al., 2022; Segen et al., 2021b). We postulated that the presence of the additional objects in the environment would improve the precision of participants’ representations of the target object location (Cánovas et al., 2011; Chamizo et al., 2011; Ekstrom & Yonelinas, 2020; Kamil & Cheng, 2001; Luo et al.,^ 2007^) as well as the understanding of the perspective shifts. Thus, we predicted smaller errors and a reduced *perspective shift-related bias* when additional spatial cues were present in the scene.

### Method

#### Participants

Forty-five young adults (mean age = 20.70 years, *SD* = 3.26; age range: 18–33 years; 25 females and 20 males) took part in this study. Participants were recruited through the participant recruitment system of Bournemouth University and received course credit for their participation. All participants gave their written informed consent in accordance with the Declaration of Helsinki (World Medical Association, 2004).

#### Design

The experiment followed a within 2 (environment: *±*) × 3 (camera translation: *left translation/no translation/right translation*) × 3 (camera rotation: *left rotation/no rotation/right rotation)* design.

#### Materials

**Fig. 3:**
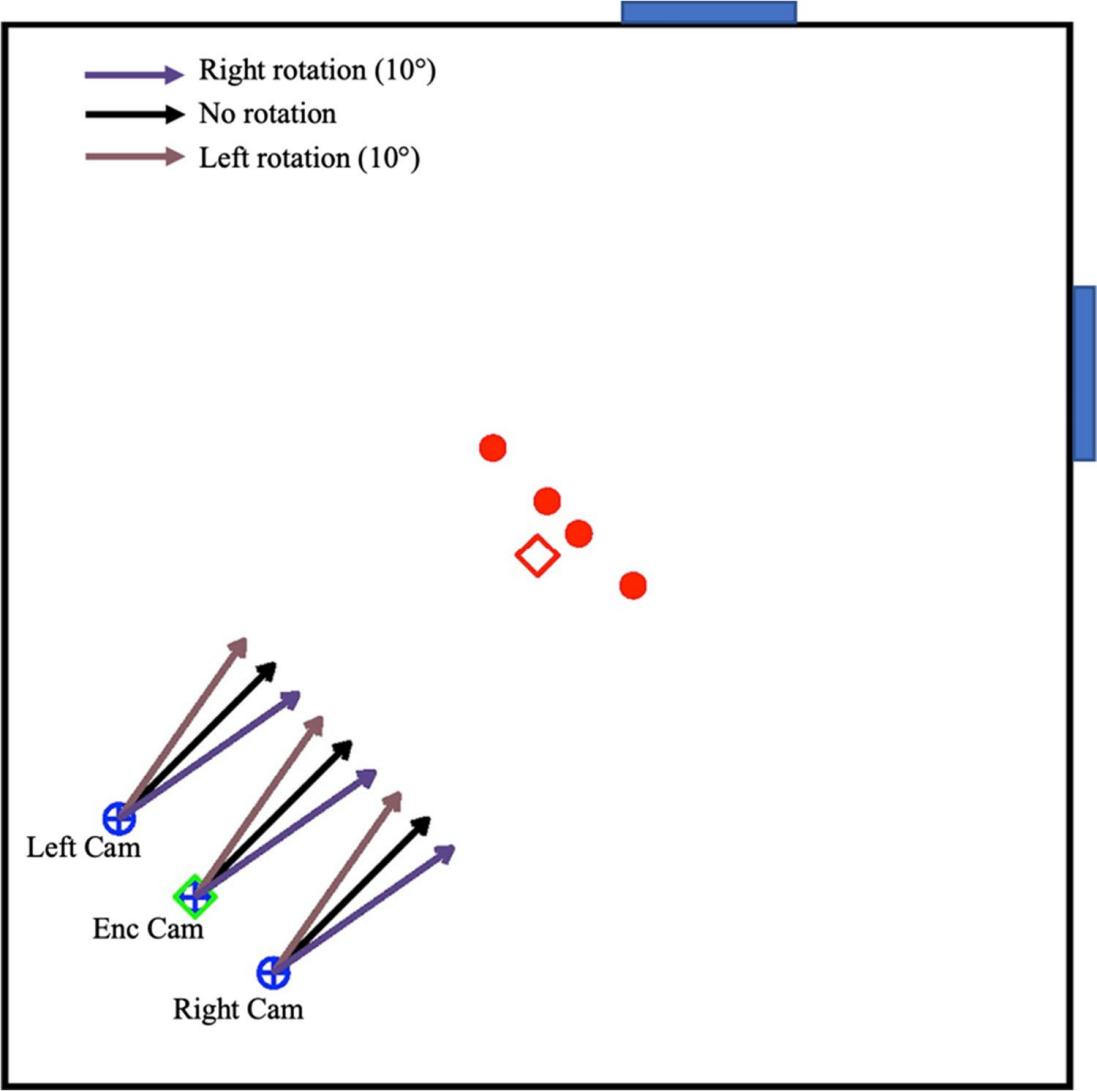
Top-down schematic of the experimental setup. Red circles indicate target object locations, with the red diamond denoting the center of the room. “Enc Cam” indicates encoding camera position, while “Left Cam” and “Right Cam” refer to test camera positions after a 1.5-m lateral shift. Arrows indicate possible camera heading directions during the test phase: right, no, or left rotation. Encoding stimuli rendered from Enc Cam with no rotation. (Colour figure online)

**Fig. 4:**
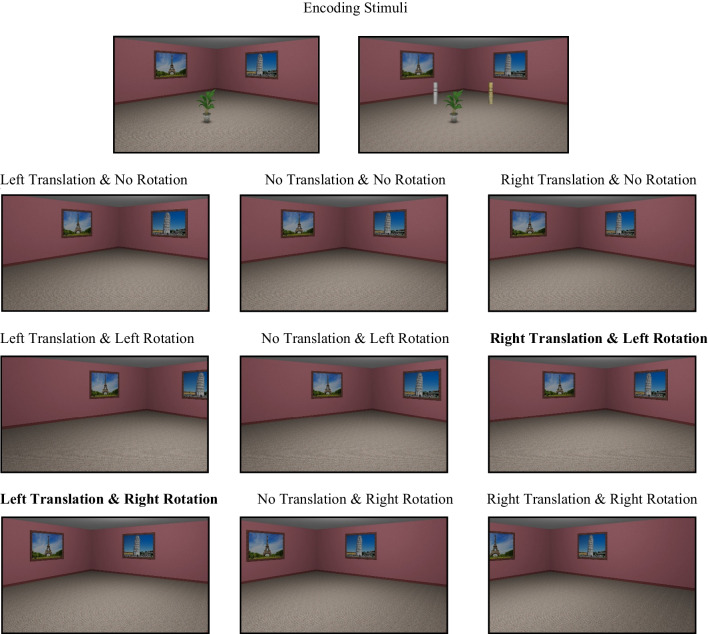
Upper image depicts sample encoding images for the Environment **−** and **+** conditions, respectively. The images below show examples of test scenes across different camera translation and rotation combinations. Bold camera translation and rotation combinations (*incongruent*) represent camera movements similar to these used in Experiment 1 and in previous studies reporting the *perspective shift-related bias* (Colour figure online)

We used the same virtual environment as in Experiment 1. In this experiment, however, we only used four predefined target object locations (14 and 28 cm to the left and to the right of centre of the diagonal line in the middle of the room), and the encoding scenes were rendered only from the central camera (Fig. 3). During encoding, the camera was oriented to face the centre of the room. For the test stimuli, the target object was removed, and the scenes were rendered from one of the three test camera locations such that the camera either remained in the same location, moved to the left, or moved to the right by 1-m from the encoding (centre) location. The rotation of the camera was also manipulated at test such that the camera either rotated by 10° to the left, 10° to the right, or did not rotate at all. This design yielded a total of nine possible combinations of camera locations and rotations for the test stimuli (Figure 4). In the Environment *+* condition, a white and yellow round column, similar to those used in previous research (Segen, Colombo, Avraamides, Slattery, & Wiener, 2021b) were added to the environment (Figure 4). These columns were added to serve as additional environmental cues.

#### Procedure

The experimental procedure was identical to that of Experiment 1. Each of the four possible target object locations was presented twice for each camera translation, rotation, and environment combination. This resulted in a total of 144 experimental trials that were preceded by two practice trials. The entire study took approximately 30 minutes to complete and was run online using Testable (testable.org).

#### Data analysis

Prior to analysis, outlier responses were removed using the interquartile range method on individual absolute distance error (m) distributions, which led to a 4.25% data loss. Data were analyzed with LMMs (R and LME4) and included environment, camera rotation, and translation as separate fixed factors. Effect coding was used for environment whilst both camera translations and rotations were coded using treatment coding with *no rotation* and *no translation* used as a baseline, respectively. A random by-subject and by-item intercept were included in the analysis. The primary aim of Experiment 2 was to investigate if camera movements systematically bias the direction of object location estimates, thus, as in Experiment 1, we have focused our analysis on signed angular error (°). The comparison between signed angular error for comparable camera movements between Experiments 1 and 2 are reported in the Supplementary Materials.

### Results

#### Signed angular error

**Fig. 5:**
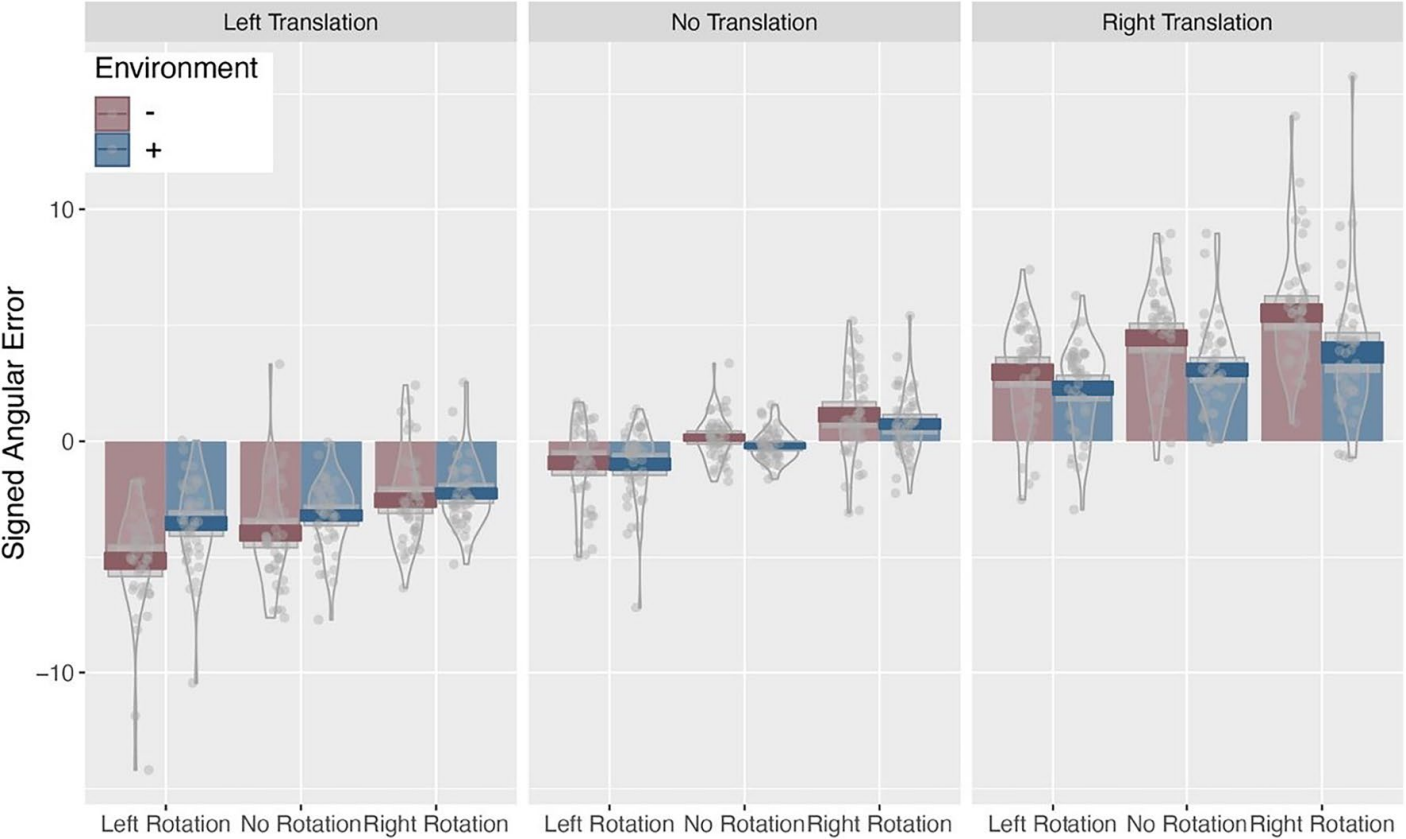
Signed angular error as a function of camera translations, rotation, and environment. Positive errors indicate that the target object was estimated to the right of the correct location, and negative errors indicate that the object was estimated to the left of the correct location. (Colour figure online)

The LMM analysis, presented in Table 1 and Fig. 5, revealed that the presence of camera rotations introduced a small bias in participants’ signed angular error in the direction of the camera rotation. Camera translations had a much larger effect, with participants’ estimates of target object locations showing a large shift in the direction of the translation. To quantify the differences between the effect of camera rotations and translations, we conducted linear hypothesis tests and found that the effect for each direction of the camera rotation was significantly different from the corresponding effect for each direction of camera translation (i.e., *left* translation vs. *left* rotations; *p* < .001). Next, we compared the magnitude of that difference and found that the effect of camera translation on signed angular error is twofold to that of camera rotations (*p* < .05). Contrary to our expectations, we did not see an effect of environment and no other significant main effects or interactions were found. For clarity, only main effects are presented in Table 1 with the full model presented in the Supplementary Materials.

**Table 1:** Partial LMM analysis of signed angular error

Predictors	Signed angular error		
	Estimates	*SE*	*t* value
(Intercept)	−0.026	0.291	−0.090
Environment (+)	−0.157	0.285	−0.551
Rotation (Left)	−0.963	0.403	**−2.391**
Rotation (Right)	0.959	0.404	**2.376**
Translation (Left)	−3.477	0.403	**−8.622**
Translation (Right)	3.765	0.403	**9.340**

*Note. t* values over 1.96 are in bold

#### Linear combination of errors for camera rotations and translations

To investigate how camera rotations and translations interact, we modelled predictions for combined movements based on rotation and translation data. Specifically, we created three models based on participants responses, (1) signed errors were solely affected by camera rotation (rotation-only model), (2) signed errors were solely affected by camera translation (translation-only model), and (3) assumed an additive influence of camera rotation and translation (additive model). Lastly, we also included a screen-based model based on predicted errors if participants used a screen frame of reference and memorized the pixel position of the target object at encoding, ignoring camera movements.

**Fig. 6:**
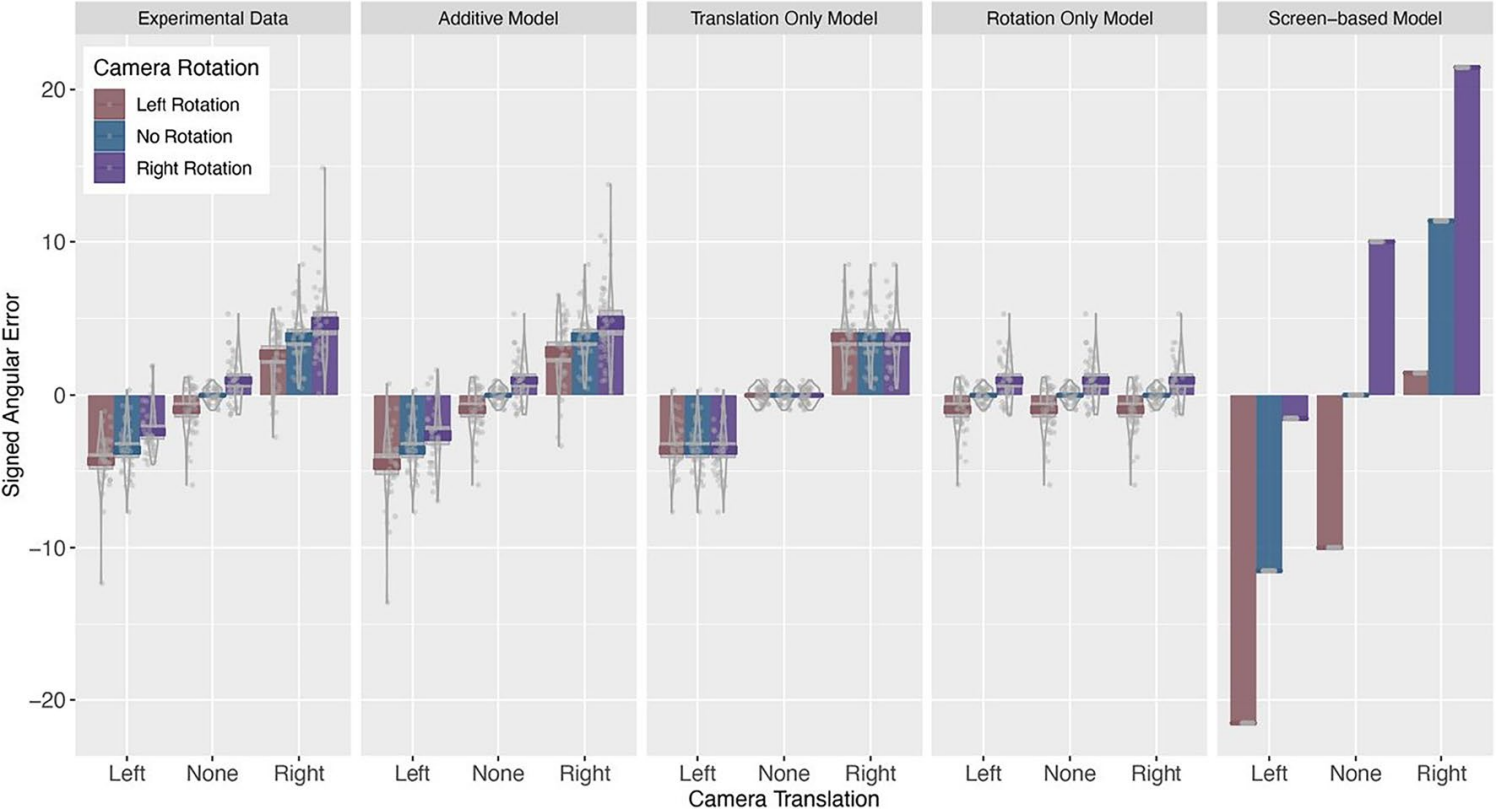
Signed angular error for experimental data and predictions of the additive, translation only, rotation only, and screen-based models. (Colour figure online)

The predictions of the four models, along with the experimental data, are presented in Fig. 6. Visually, it is apparent that participants’ errors are unlikely to be driven solely by camera rotations, nor are they using the screen-based strategy. On the other hand, both the translation-only model and the additive model fit the experimental data well. However, the additive model provides a significantly better fit than the translation-only model (translation only RSS = 862.61, additive model RSS = 373.58, *F* = 526.22, *p* < .001). The close fit of the predictions of the additive model for the combined camera movements with the actual data suggests that camera rotation and camera translation independently influence participants’ performance.

### Discussion

In the present study we investigated the role that camera rotations and translations have on the errors participants make when recalling object locations. We also examined whether enriching the environment by providing additional spatial cues reduces the *perspective shift-related bias*. Importantly we replicated the *perspective shift-related bias* we described in our previous studies (Segen et al., 2022; Segen et al., 2021a, 2021b) and in Experiment 1. Specifically, we found that participants’ responses were biased in the direction of camera movements for both rotations and translations, yet this bias was stronger with the introduction of camera translations, compared with rotations. Contrary to our predictions and previous work (Segen et al., 2021b), we did not see a reduction in the bias introduced by camera movements with the presence of additional spatial cues in the scene. However, we observed a trend suggesting that the effect of camera translations on signed angular error was lower in the presence of additional environmental cues. Notably, this interaction was significant in raw data, in which outliers were not removed. See Supplementary Materials for analyses without the removal of outliers. It is possible that the addition of only two uniform columns in the scene was insufficient to reliably reduce uncertainty with camera translations and therefore yielded only a small (and unreliable) benefit for resolving camera translations. Future investigations with more informative environmental cues are needed to determine if increasing the number of spatial cues in the environment could reduce the bias in object location estimates.

We propose that the differential effects of camera rotations and translations on participants’ performance are driven by differences in how camera rotations and translations affect the egocentric self-to-object relations and the 2D projections of object-to-object relations. To estimate object locations following a perspective shift, participants need to (1) encode the location of the target object, (2) compare the encoding and test scenes to understand how they have moved through space (i.e., to understand the perspective shift which requires self-localization at both encoding and test), and, finally, (3) recompute the target object location given their new location in the environment. When camera rotations are introduced, the distance to the object and other features in the environment remain constant but the location of the object and other features of the environment on the screen are uniformly offset by the rotation angle. Thus, the relative location of the target object in relation to other features in the environment on the image remains the same despite appearing at a different part of the image. As a result, participants do not need to self-localize after camera rotations as they can rely on their memory for the object’s location relative to other nearby features in the environment, similarly to a snapshot memory model (Franz et al., 1998). Alternatively, they can use the offset in the location of other features in the environment to estimate the location of the target object. However, when camera translations are introduced, the distance between one’s own location and other objects changes. Notably, this change is not uniform and depends on the location of the objects within the environment. This leads to changes in the vectors and angles between the self and the environmental features, including the to-be-remembered object locations and the locations these features occupy on the screen. Participants need to consider this new information to understand how they moved through space, and to update the target object’s location accordingly.

Since camera translations are more difficult to resolve than camera rotations, they introduce more uncertainty about the location of the target object. Consistent with the anchor and adjustment heuristic (Tversky & Kahneman, 1974), we suggest that due to higher uncertainty following camera translations than rotations, participants rely on an egocentric anchor (Epley et al., 2004; Gilovich et al., 2000; Keysar et al., 2000). According to the anchor and adjustment heuristic, the anchor is typically adjusted until a plausible response is reached, However, such adjustments are often insufficient (Quattrone, 1982; Tversky & Kahneman, 1974) such that the response remains biased in the direction of the initial estimate. In our task, the egocentric anchor is the self-to-object vector during encoding. Insufficient adjustment of this egocentric vector on the basis of the perspective shift would result in a systematic shift in object location estimates in the same direction as camera translations and rotations. And since camera translations result in greater uncertainty and consequently greater reliance on the anchor, the systematic shift is greater when camera translation rather than camera rotations, are introduced.

An alternative explanation for the larger bias towards camera translations rather than rotations is that rotations may provide a “smaller” spatial manipulation, causing participants to fail to detect it. If participants fail to detect a camera rotation, we would expect them to place the object in the same (pixel) position as seen during encoding, which would yield larger signed angular errors, similar to pure translations, as can be seen for the error prediction for screen-based strategy. However, since participant errors do not follow this pattern, we believe that they are able to detect the camera manipulations and are not relying on a 2D screen-based strategy to solve the task. It should be noted, however, that we did not match the amount of “change” introduced by the camera rotation and translations as they are qualitatively different and difficult to compare. Instead, we select the maximum rotation and translation that enables all camera movement combinations while maintaining visibility of all spatial cues during both encoding and test phases. Future studies should consider systematically manipulating camera rotations and translations to investigate how this influences the presence of the systematic bias in the direction of the camera movement.

The finding in our study of a greater detrimental effect of camera translations than rotations on overall performance and on the systematic bias in object location estimate is inconsistent with findings from the spatial updating literature that typically show imagined rotations to have a more debilitating effect on performance than imagined translations (Klatzky, 1998; Presson & Montello, 1994; Rieser, 1989; Wraga, 2003). Greater error is observed during imagined rotations than translations because the latter are less computationally demanding (Presson & Montello, 1994; Rieser, 1989). For example, Rieser (1989) argued that during imagined translations participants can simply retrieve the stored information from memory. For imagined rotations, however, participants either need to recompute the object-to-object relations taking into account their new orientation or combine the signed self-to-target angle and the signed self-to-observation point angle. Both strategies require additional mental computations to transform the initial encoded representation of object locations.

Presson and Montello (1994) suggested that differences between the imagined rotations and translations in a spatial updating task may be driven by a conflict between actual and imagined heading directions. Specifically, they proposed that we have a strong tendency to use our immediate heading direction as a primary frame of reference. And in the imagined rotation condition participants need to override this primary frame of reference to adopt an alternative imagined heading direction. Such conflict between reference frames is not present in the translation condition as the actual and imagined heading always remain the same. The lack of conflict between reference frames is also likely to make the updating of self-to-object relations easier (Presson & Montello, 1994).

In our task, however, the impact of camera rotations and translations is different. Specifically, the object-to-object relations as projected on the screen change in the camera translation condition but not in the camera rotation condition. In addition, as noted earlier, the self-to-object relations are uniformly offset in the rotation condition, therefore the new self-to-target object relations can be calculated much easier in conditions when camera rotations are introduced. In the translation condition, on the other hand, participants need to engage in more demanding computations to estimate the new self-to-target object location. Furthermore, in our task, there is no conflict between heading directions. Participants are shown their new heading direction instead of imagining it. Therefore, their new heading is apparent in both encoding and test phases. Thus, in our view, the differential impact of rotations and translations between our task and the spatial updating paradigms is responsible for differences in the results.

The experimental design we employed allowed us to investigate how camera rotations and translations combine to influence participants’ performance. We found that a simple linear model with additive inputs of pure rotation and pure translation errors closely matches the empirical data for combined camera movements and provides a significantly better fit than models that are based on errors associated with translations or rotations only. This result suggests that rotation and translation influences do not follow the winner-takes-it-all principle that has been used to explain higher-level cognitive phenomena such as visual attention (Itti & Koch, 2001; Walther & Koch, 2006) and decision making (Furman & Wang, 2008; Wang, 2002). Instead, we believe that performance on trials with combined camera translation and rotations results from independent influences of rotations and translations that are linearly combined to produce the observed errors. The linear additive model also explains the smaller errors observed after incongruent camera movements (camera rotates and translates in opposite direction) compared with congruent camera movements (camera rotates and translates in the same direction). Specifically, in incongruent movements, the errors have opposite signs since they are biased in the direction of movement for both camera rotations and translations. Therefore, when the errors are combined, they cancel each other out. In congruent movements, the errors for rotations and translations are biased in the same direction and are therefore additive.

Lastly, it is important to consider a potential limitation in the methodology of Experiment 2 compared with Experiment 1. In Experiment 1, we aimed to establish the new task and replicate the perspective shift-related bias reported in our earlier research (Segen et al., 2022; Segen et al., 2021a, 2021b). In Experiment 2, we reduced the number of possible target positions from 18 to four and limited the number of camera positions for rendering encoding stimuli to one. The reasons for these changes were twofold: (1) We introduced more experimental manipulations, so to match the same number of object positions and camera positions would have required 648 trials. To reduce the experiment’s duration, we limited the number of unique encoding stimuli. (2) Some combinations of camera translation and rotation would have resulted in stimuli where the two posters were outside the frame, so we avoided using object and encoding camera positions that were further away from the center of the room.

It is possible that by reducing the number of to be remembered object positions, we altered the demands of the task and reduced the uncertainty regarding the target object’s position, which could have influenced participants’ responses to the introduced camera manipulations. A direct comparison of signed angular errors in Experiments 1 and 2 for similar camera translations and rotations suggests that we were able to replicate the same effect—that is, errors are biased towards the perspective shift direction (as defined in Experiment 1); however, error magnitudes were lower (Supplementary Materials). Given that we can replicate the bias and see variability in the bias as a function of camera rotations and translations, we are confident that the results reported in Experiment 2 are driven by the same processes that gave rise to the results in Experiment 1 and our previous work. Furthermore, the reduction in error magnitudes in Experiment 2 (fewer object positions) aligns with the idea that uncertainty mediates the reliance on the egocentric anchor, causing participants to rely less on it and potentially improving the adjustment process. Specifically, having a limited number of object locations (closer to the center of the room) limits the range of plausible object locations participants select during the adjustment process. Of note, it is possible that we do not see an effect of additional environmental cues on reducing the observed bias, due to already relatively small angular errors and the improved adjustment process due to limited number of possible object positions. Future studies should investigate if by increasing the task demands, presence of additional objects contributes to the reduction of the bias in object location estimates in the presence of camera movements.

## General summary

To summarize, in the present study we evaluated people’s ability to estimate object positions following a perspective shift. In Experiment 1, we replicated (Segen et al., 2022; Segen et al., 2021a) a systematic shift in position estimates in the same direction as the perspective shift. In Experiment 2, we investigated the contribution of camera rotations and translations to this bias and showed that translations are largely responsible for driving the systematic bias in object location estimation. Camera translations introduced greater change in the relations between the own location and the object as well as other features in the environment compared with rotations. We believe that those greater changes lead to increased uncertainty regarding the location of an object in the environment which results in greater reliance on egocentric anchors leading to the systematic bias in errors in the same direction as translations. To our knowledge, this is the first study to show that the influence of camera rotations and translations on participants’ performance is guided by a linear additive process.

### Supplementary information

ESM 1 (DOCX 251 kb)
