# Supplementary material for: Biases in object location estimation: The role of rotations and translation
Source: Atten Percept Psychophys. 2023 May 31;85(7):2307–20. doi: 10.3758/s13414-023-02716-2 (PMC10584736; doi:10.3758/s13414-023-02716-2)
Supplement: Supplementary file 1 — (DOCX 251 kb) [file 13414_2023_2716_MOESM1_ESM.docx]

**Absolute distance error analysis for Experiment 1**

Absolute distance errors are defined as the distance between the correct object position and the position that participants chose. An LMM analysis with Perspective shift direction (PSD) and Object Position as a fixed effect revealed that overall, the distance between the location participants chose and the correct position was 1.00m (Intercept: β=1.003, SE=0.031, t=32.637). These distance errors were not affected by PSD or Object Position.


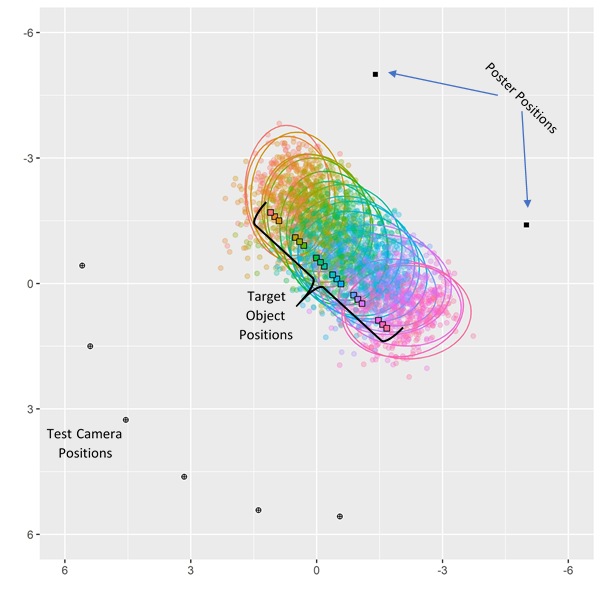


Figure S1 Scatter plot of participants' responses as a function of target object positions. Target object positions are depicted using multi-coloured squares, with each colour representing a different target object position, points that correspond in colour to the squares represent participants' responses for the given target object with error ellipses.

As apparent from Figure S1 participants’ systematically overestimated the distance of the object. To quantify this effect we subtracted the distance between the correct object position and camera position (i.e. the position from which the stimulus was rendered) from the distance between the chosen position and the camera position (on the y-axis). An LMM analysis with PSD and Object Position as a fixed effect revealed that participants overestimated the distance of the object thereby placing it further away from the correct position (*Intercept*: β=0.452, SE=0.050, t=9.088). This overestimation was not affected by PSD or Object Position.

We believe that this overestimation is driven by participants not using the base of the target object when reporting its position i.e., they might be using a higher part of the object as their reference point. To test that, we calculated predicted responses if participants used the centre of the plant pot as their reference point for the target object, and found that the average distance overestimation was 43.17 cm which is very similar to the amount of overestimation we observed in our results (45.20 cm).

Consequently, we propose that the systematic distance overestimation arose because participants did not use the base of the object when encoding and then estimating its position after the perspective shift. Specifically, during the analysis, we assume that participants indicate the position of the object using the base as a reference point and by clicking on the floor where the base would be. However, if participants do not use the base of the object but instead use a part of the object that is higher up, such as the centre of the plant pot, this would lead participants to click on a floor location that is systematically further away from the position of the virtual camera.

**Absolute distance error analysis for Experiment 2**

Table 1 LMM coefficients for participants Absolute distance error (m)

|  | Absolute distance error (m) | | |
| --- | --- | --- | --- |
| *Predictors* | *Estimates* | *std. Error* | *t-value* |
| (Intercept) | 0.647 | 0.042 | 15.510 |
| Environment (+) | -0.103 | 0.016 | -6.583 |
| Rotation (Left) | 0.051 | 0.022 | 2.296 |
| Rotation (Right) | 0.049 | 0.022 | 2.185 |
| Translation (Left) | 0.108 | 0.023 | 4.615 |
| Translation (Right) | 0.129 | 0.023 | 5.504 |
| Environment (+) * Rotation (Left) | 0.014 | 0.022 | 0.645 |
| Environment (+) * Rotation (Right) | -0.014 | 0.022 | -0.608 |
| Environment (+) * Translation (Left) | -0.007 | 0.022 | -0.310 |
| Environment (+) * Translation (Left) | -0.012 | 0.022 | -0.562 |
| Rotation (Left) * Translation (Left) | 0.001 | 0.031 | 0.036 |
| Rotation (Left) * Translation (Right) | -0.079 | 0.031 | -2.527 |
| Rotation (Right) * Translation (Left) | -0.097 | 0.031 | -3.101 |
| Rotation (Right) * Translation (Right) | -0.013 | 0.032 | -0.422 |
| Environment (+) * Rotation (Left) * Translation (Left) | -0.022 | 0.031 | -0.699 |
| Environment (+) * Rotation (Right) * Translation (Left) | 0.012 | 0.031 | 0.374 |
| Environment (+) * Rotation (Left) * Translation (Right) | -0.000 | 0.031 | -0.002 |
| Environment (+) * Rotation (Right) * Translation (Right) | 0.007 | 0.032 | 0.238 |

**Signed angular error full model from Experiment 2**

Table 2 LMM coefficients for participants Signed angular error (°)

|  | Signed angular error | | |
| --- | --- | --- | --- |
| *Predictors* | *Estimates* | *std. Error* | *t-value* |
| (Intercept) | -0.026 | 0.283 | -0.092 |
| Environment (+) | -0.169 | 0.207 | -0.817 |
| Rotation (Left) | -0.964 | 0.392 | -2.459 |
| Rotation (Right) | 0.961 | 0.393 | 2.448 |
| Translation (Left) | -3.477 | 0.392 | -8.864 |
| Translation (Right) | 3.765 | 0.392 | 9.603 |
| Environment (+) * Rotation (Left) | 0.315 | 0.227 | 1.390 |
| Environment (+) * Rotation (Right) | -0.143 | 0.227 | -0.632 |
| Environment (+) * Translation (Left) | 0.532 | 0.227 | 2.350 |
| Environment (+) * Translation (Left) | -0.490 | 0.227 | -2.159 |
| Rotation (Left) * Translation (Left) | 0.171 | 0.555 | 0.309 |
| Rotation (Left) * Translation (Right) | 0.214 | 0.555 | 0.386 |
| Rotation (Right) * Translation (Left) | -0.194 | 0.555 | -0.350 |
| Rotation (Right) * Translation (Right) | -0.093 | 0.556 | -0.167 |
| Environment (+) * Rotation (Left) * Translation (Left) | -0.022 | 0.031 | -0.699 |
| Environment (+) * Rotation (Right) * Translation (Left) | 0.012 | 0.031 | 0.374 |
| Environment (+) * Rotation (Left) * Translation (Right) | -0.000 | 0.031 | -0.002 |
| Environment (+) * Rotation (Right) * Translation (Right) | 0.007 | 0.032 | 0.238 |

#### **Signed angular error analysis with the raw data (with outliers)**

Table 3 LMM coefficients for raw participants Signed angular error (°)

|  | Signed Angular Error | | |
| --- | --- | --- | --- |
| *Predictors* | *Estimates* | *std. Error* | *t-value* |
| (Intercept) | -0.067 | 0.287 | -0.232 |
| Environment (+) | -0.205 | 0.210 | -0.976 |
| **Rotation (Left)** | **-0.961** | **0.397** | **-2.419** |
| **Rotation (Right)** | **0.971** | **0.398** | **2.438** |
| **Translation (Left)** | **-3.597** | **0.397** | **-9.049** |
| **Translation (Right)** | **3.898** | **0.397** | **9.806** |
| Environment (+) * Rotation (Left) | 0.319 | 0.230 | 1.390 |
| Environment (+) * Rotation (Right) | -0.074 | 0.230 | -0.324 |
| **Environment (+) * Translation (Left)** | **0.599** | **0.230** | **2.610** |
| **Environment (+) * Translation (Left)** | **-0.470** | **0.230** | **-2.045** |
| Rotation (Left) * Translation (Left) | 0.138 | 0.562 | 0.246 |
| Rotation (Left) * Translation (Right) | 0.272 | 0.563 | 0.483 |
| Rotation (Right) * Translation (Left) | -0.201 | 0.563 | -0.357 |
| Rotation (Right) * Translation (Right) | 0.091 | 0.563 | 0.161 |

#### **Signed angular error for incongruent trials**


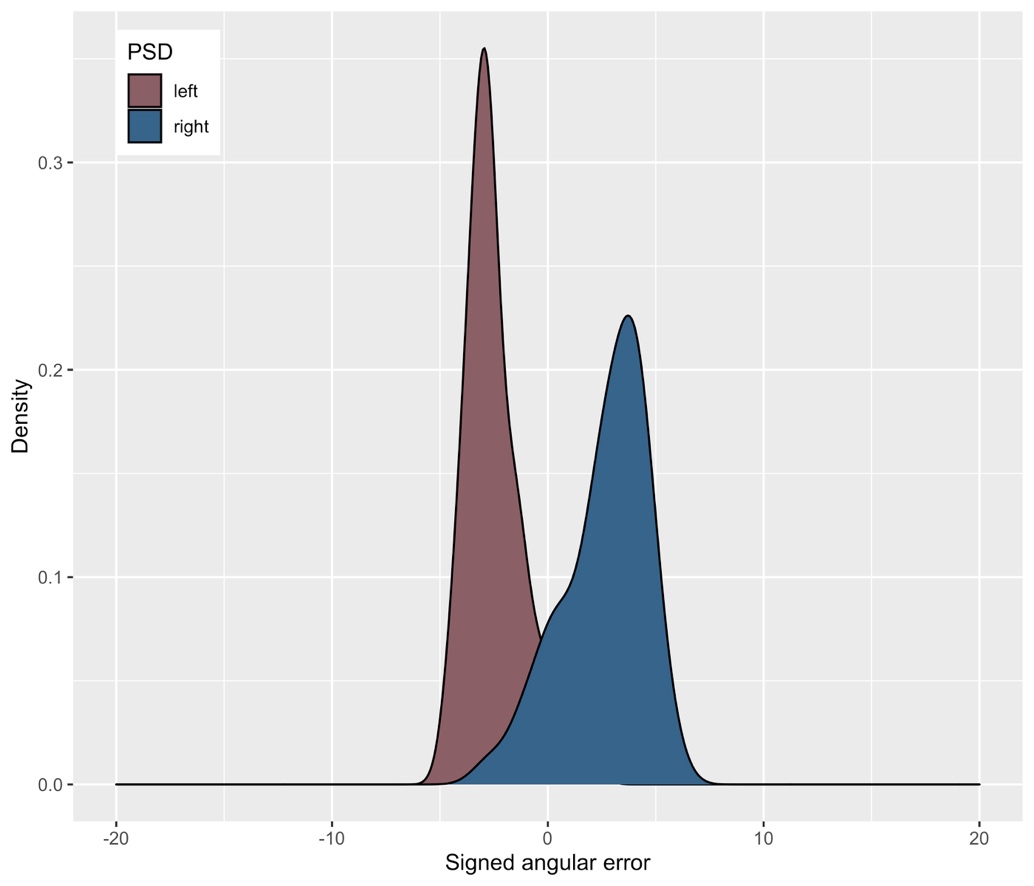


Figure S2 Signed angular error for *incongruent* camera movements (left rotation/right translation, right rotation/left translation)
